# Supplementary material for: Infinitesimal sulfur fusion yields quasi-metallic bulk silicon for stable and fast energy storage
Source: Nat Commun. 2019 May 28;10:2351. doi: 10.1038/s41467-019-10289-8 (PMC6538609; doi:10.1038/s41467-019-10289-8)
Supplement: Supplementary file 1 — Supplementary Information [file 41467_2019_10289_MOESM1_ESM.pdf]

Supplementary Information

Infinitesimal Sulfur Fusion Yields Quasi-Metallic Bulk Silicon for Stable and Fast Energy Storage

Ryu et al.

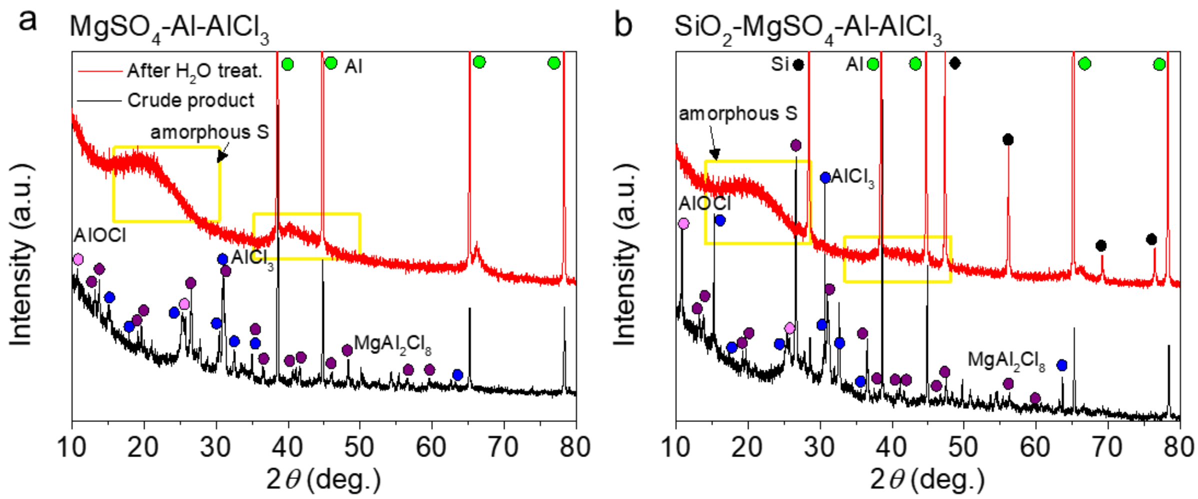

Supplementary Figure 1 | Seed formation analysis. **a, b** XRD patterns of crude products and samples after H_2O wash in (**a**) $MgSO_4$-Al-$AlCl_3$ and (**b**) SiO_2-$MgSO_4$-Al-$AlCl_3$ systems with other reaction parameters kept identical as described in the experimental section.

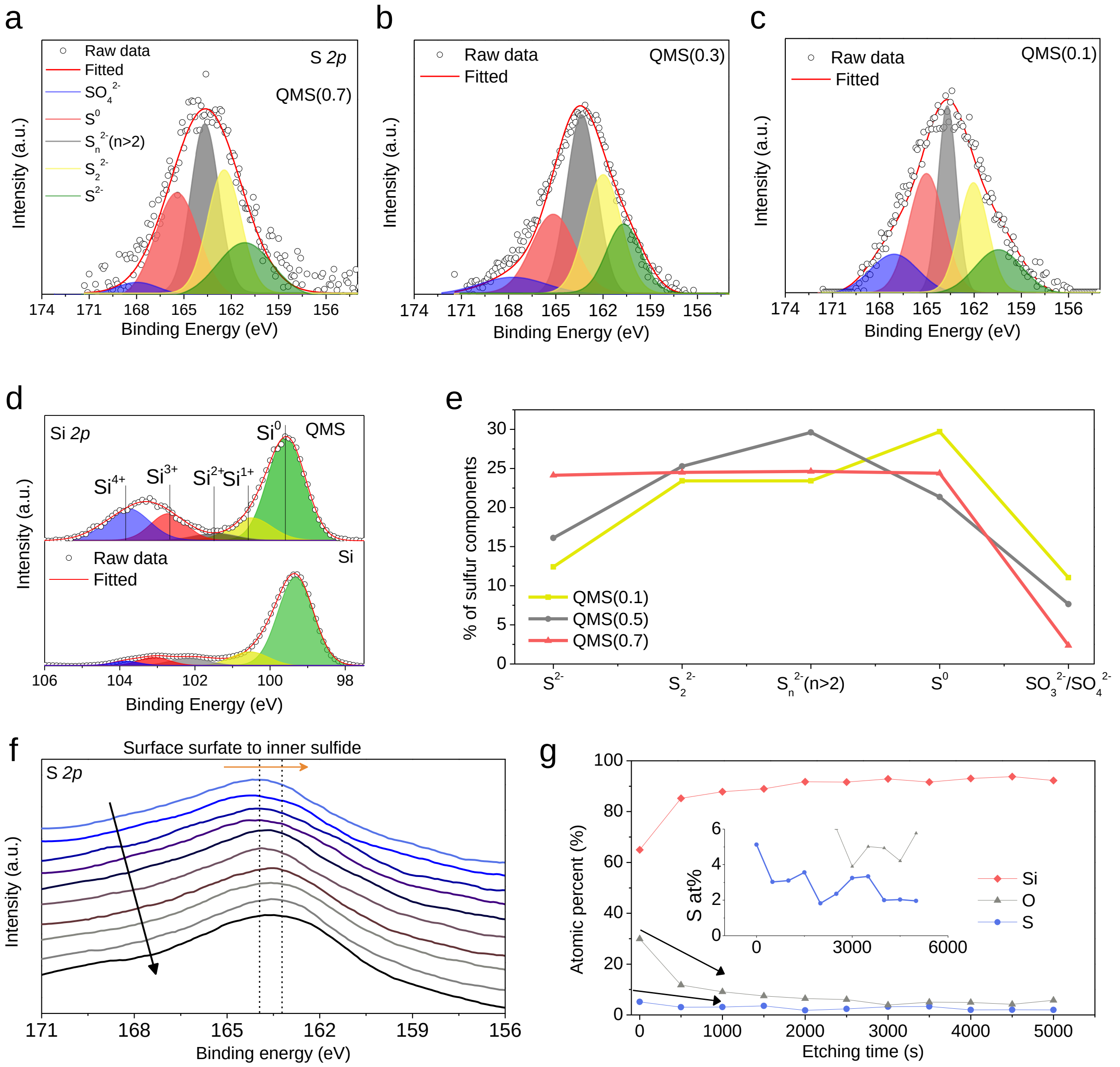

Supplementary Figure 2 | Fused sulfur state analysis. a-c X-ray Photoelectron spectroscopy results of S 2*p* core-level spectra of (**a**) QMS(0.7), (**b**) QMS(0.3) and (**c**) QMS(0.1). **d** Si 2*p* core-level spectrum of Si and QMS(0.7). **e** Proportion plot of each S component in individual QMS samples. **f** Depth-profiles of S 2*p* core-level spectrum at each 50 nm for 10 times in QMS(0.7). **g** Corresponding atomic composition of QMS(0.7) in each depth profile. The sulfur fusion induced the peak shifts assigned to Si^0 and Si^{4+}, respectively from 99.4 and 103.2 eV to 99.6 and 103.7 eV that was usually observed in doped Si materials (QMS denotes quasi-metallic silicon and following figures in the parentheses denotes the doping concentration in atomic percent).

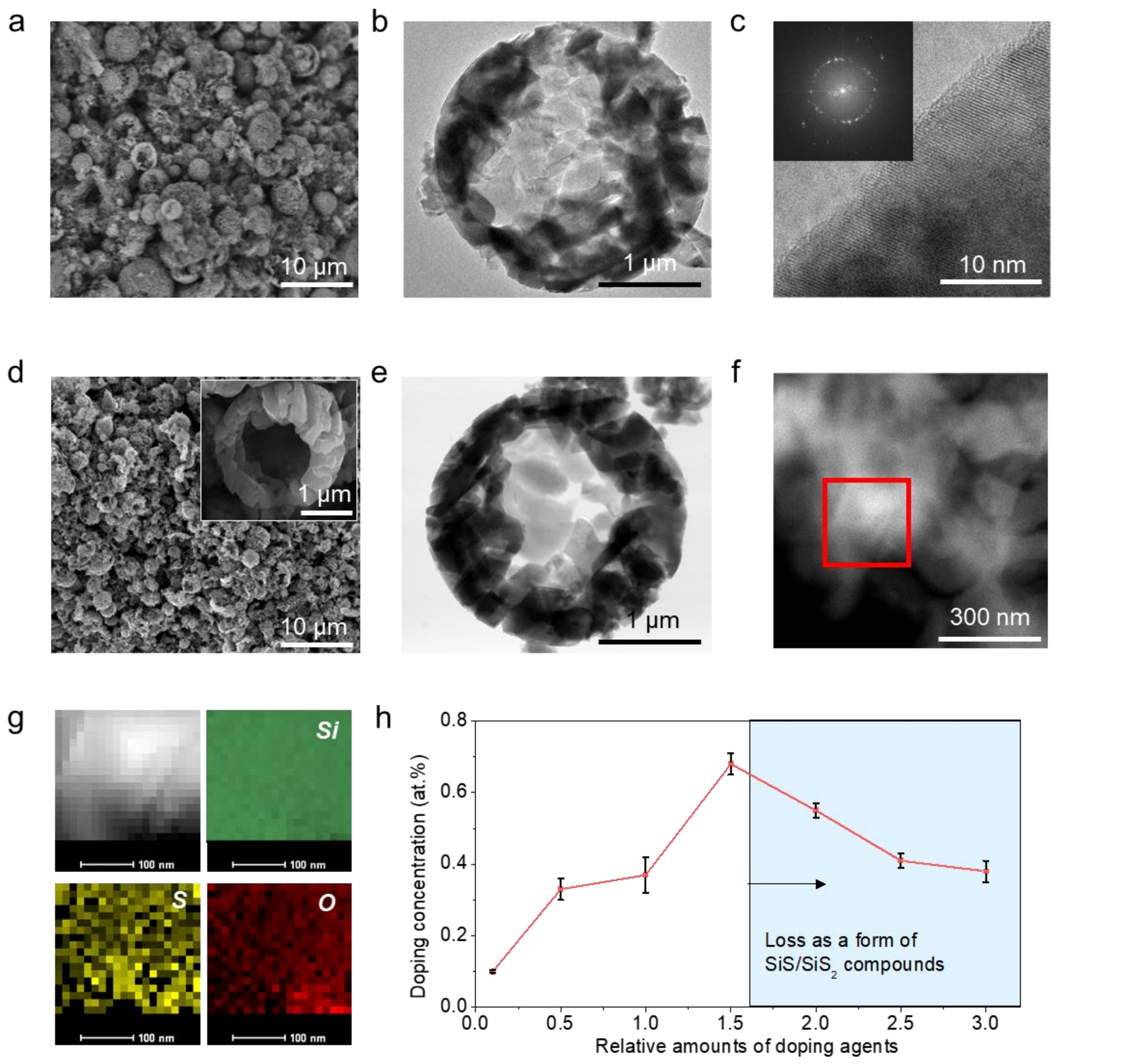

Supplementary Figure 3 | Structural characterization of Si and quasi-metallic silicon. a-c SEM image (**a**), TEM image (**b**) and High-magnification TEM image of Si (**c**), inset shows the FFT image of typical polycrystalline Si. **d-h** SEM image (**d**), inset shows high-magnification SEM image, Bright-field (BF)-TEM image (**e**), STEM image (**f**), Corresponding EDS maps (**g**) and Sulfur concentrations control results of QMS (**h**). Scale bars, 10 μm (**a, d**); 1 μm (**b, e, inset of d**) 10 nm (**c**); 300 nm (**f**); 100 nm (**g**).

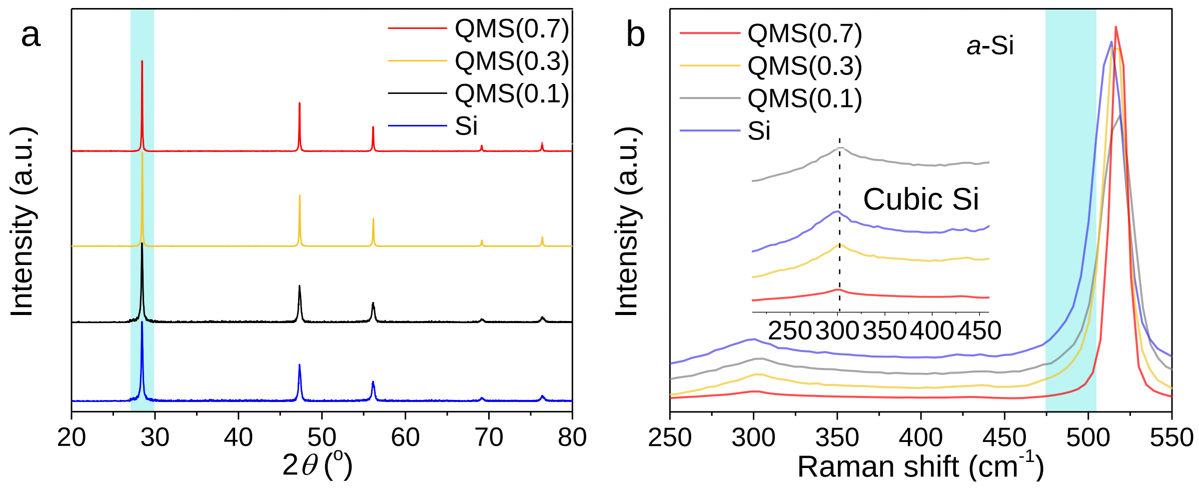

Supplementary Figure 4 | Crystalline phase analysis of Si and quasi-metallic silicon. a X-ray diffraction patterns (XRD) of Si and a series of QMS between 20–80°. **b** Raman of Si and series of QMS spectra, inset shows enlarged spectra between 220–460 cm^{-1}. Each pattern has identical characteristic peaks of crystalline Si and (111) peaks are all shifted to a lower angle, thus suggesting the expansion of lattice structure due to chain-like sulfur incorporation, unlike entire substitutional doping. The cubic crystalline phase of Si was confirmed through Raman spectroscopy without a trace of other polymorphs that can be generally observed from strong light-matter interactions during ion-implantation.

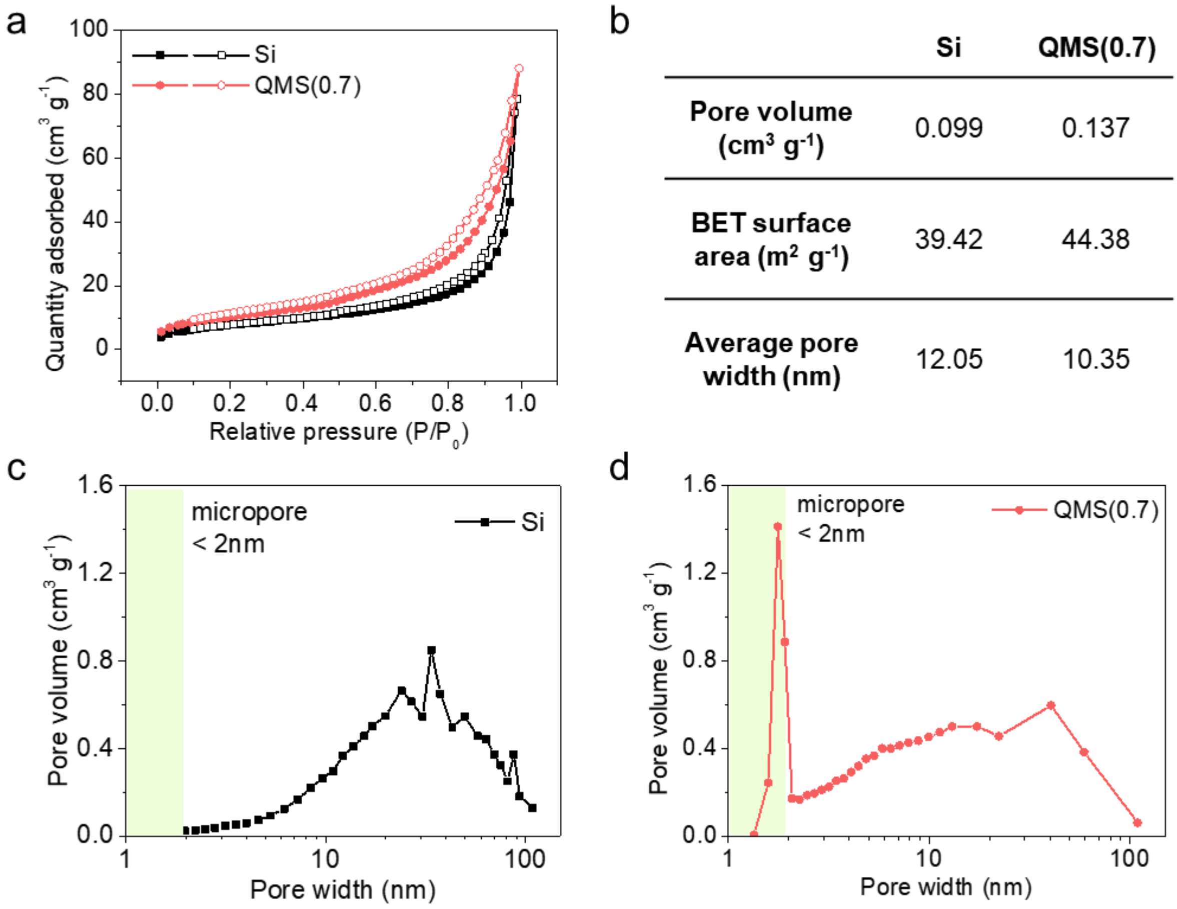

Supplementary Figure 5 | Porous structure analysis of Si and quasi-metallic silicon. a-d, Nitrogen adsorption and desorption isotherms (**a**), Summarized values for pore volume, BET surface area and average pore width (**b**), Pore size distribution curve of Si (**c**) and QMS(0.7) (**d**)**.** Compared with typically prepared porous silicon materials (0.5–2.0 cm^3 g^{-1}), this low-temperature reduction process gives a moderate level of pore volume and surface area to avoid high amounts of irreversible capacity at the initial cycles.

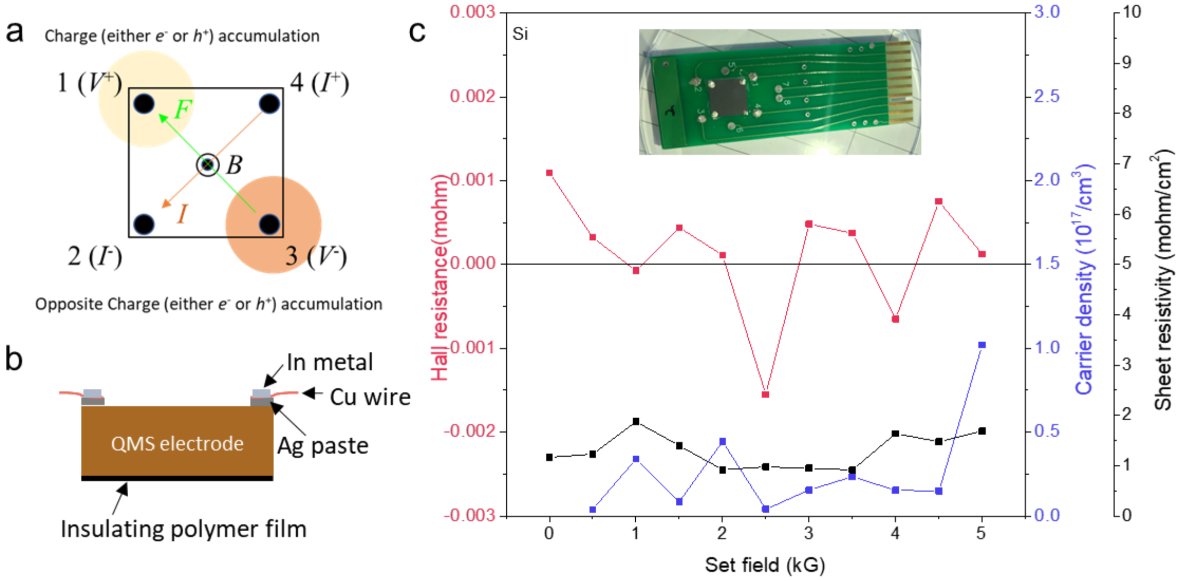

Supplementary Figure 6 | Hall effect measurement. a-c Fundamental principle of hall effect measurement under specific direction of magnetic field and voltage (**a**), Schematic illustration of the experimental setup for hall measurement (**b**) and Hall resistance, carrier density, and sheet resistivity plots of undoped Si versus set fields (inset. digital photograph of experimental setup) (**c**).

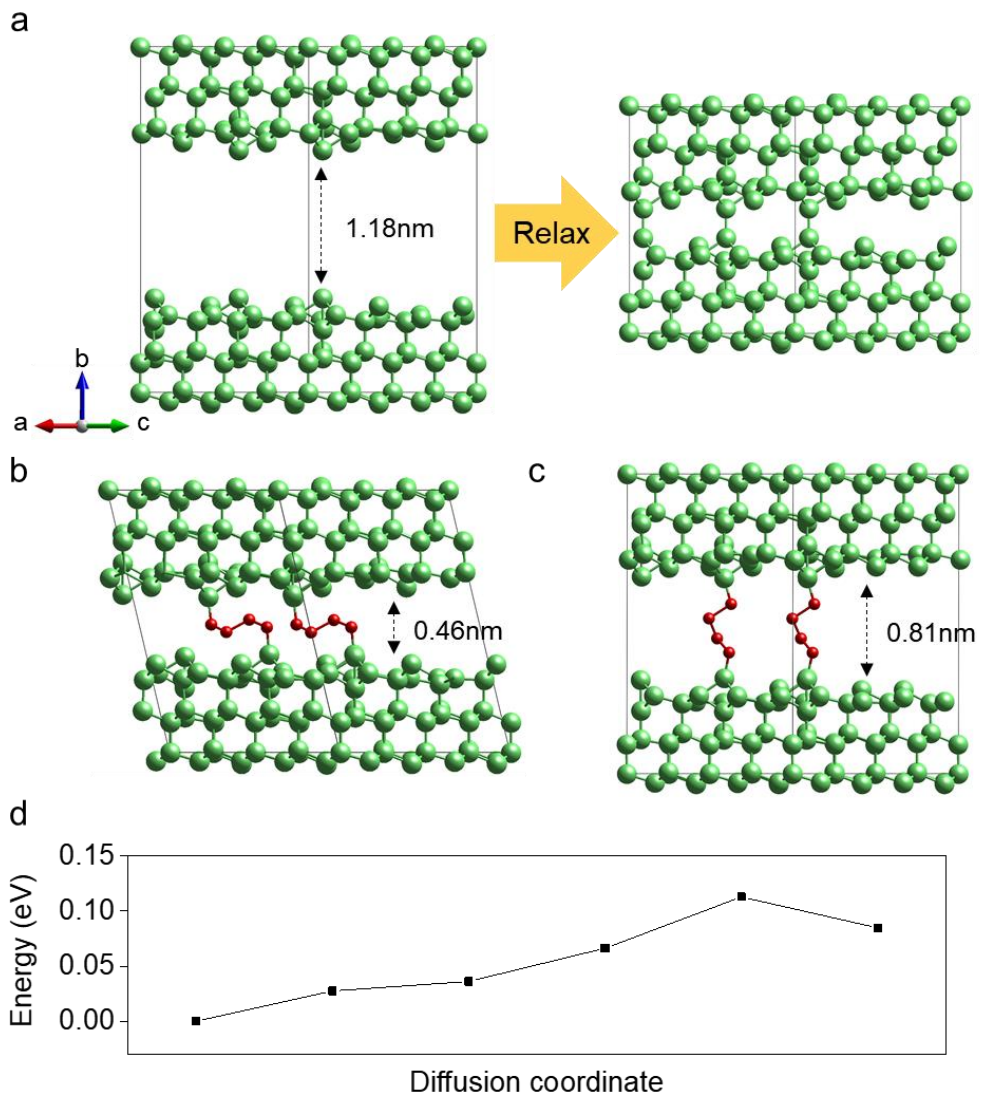

Supplementary Figure 7 | The channel structure of Si (111) slab with sulfur chains and Li migration energy barrier calculated by DFT. a Initial channel structure with slab spacing of 1.18 nm (left) and its fully-relaxed channel structure without sulfur chains (right). **b** Fully-relaxed structure with the slab spacing of 0.46 nm from initial spacing range of 0.51 to 0.70 nm with sulfur chains. **c** Fully-relaxed structure with the slab spacing of 0.81 nm from initial spacing range of 0.80 to 0.99 nm. **d** Diffusion barrier of Li-ion through the channel center.

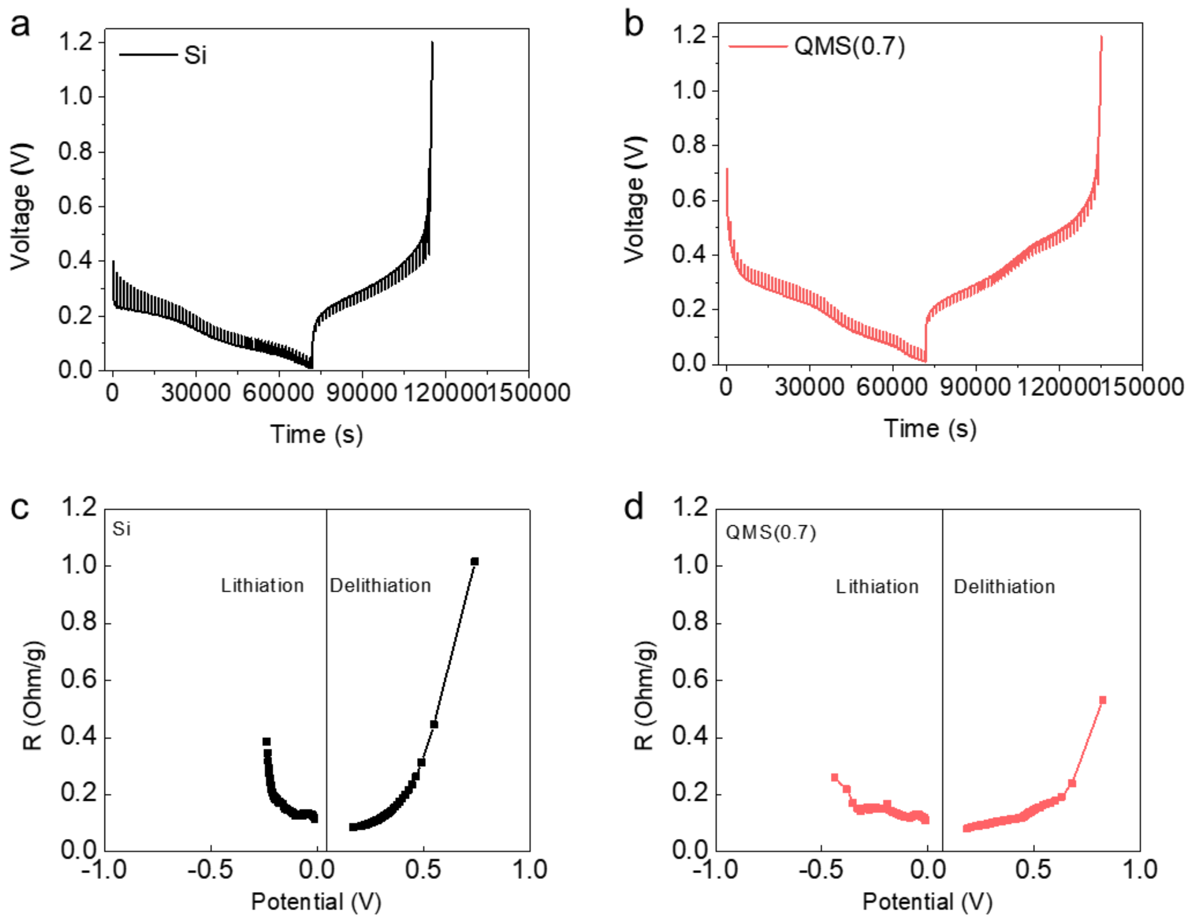

Supplementary Figure 8 | GITT measurement of quasi-metallic silicon and Si electrodes. **a, b** The GITT profiles of (**a**) Si and (**b**) QMS(0.7) electrodes during the first lithiation/delithiation process. **c, d** The variation of the internal cell resistance determined from the (**c**) Si and (**d**) QMS electrodes during the lithiation/delithiation process.

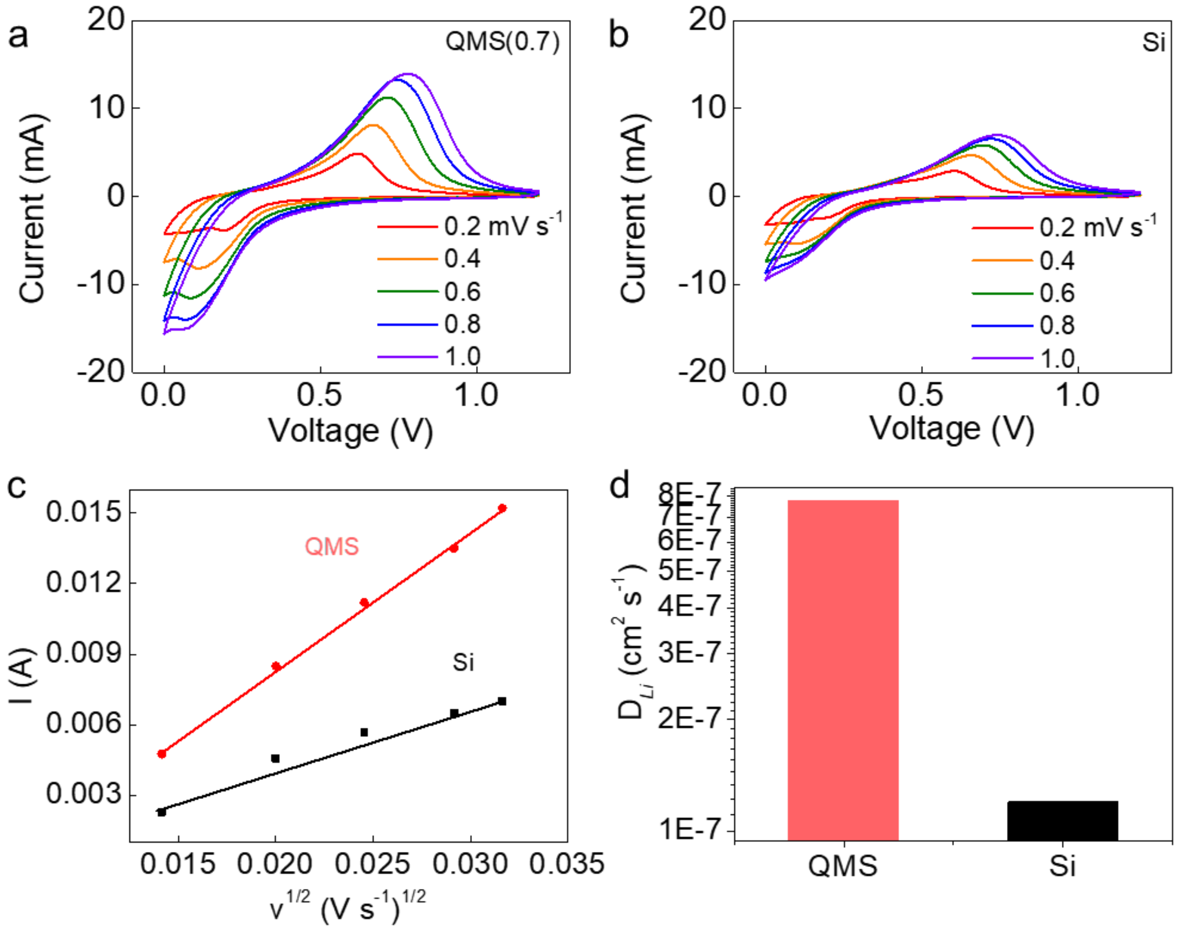

Supplementary Figure 9 | Current responses during CV measurements. a, b Cyclic voltammograms for (**a**) QMS(0.7) and (**b**) Si electrodes at different scan rates from 0.2 to 1.0 mV s^{-1}. **c** The fitting results of the reduction peak current versus the square root of the scan rates for the QMS(0.7) and Si electrodes at the early cycles. **d** Calculated diffusion coefficient (D_{Li}) after CV measurement that corresponds to the state after 50 cycles.

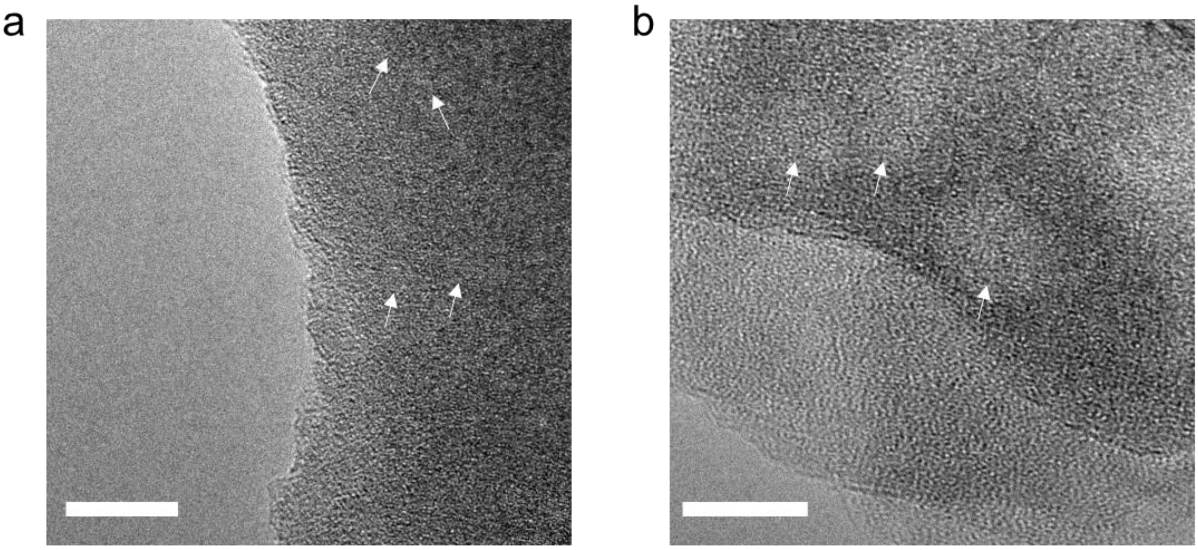

Supplementary Figure 10 | Post-cycle TEM analysis after SEI removal. a, b TEM images showing (**a**) micropores and (**b**) mesopores that are assumed to be the trace of etched lithium sulfide during acid treatment to remove SEI layer in harsh conditions for QMS(0.7) electrode. Scale bars, 20 nm (**a**); 10 nm (**b**).

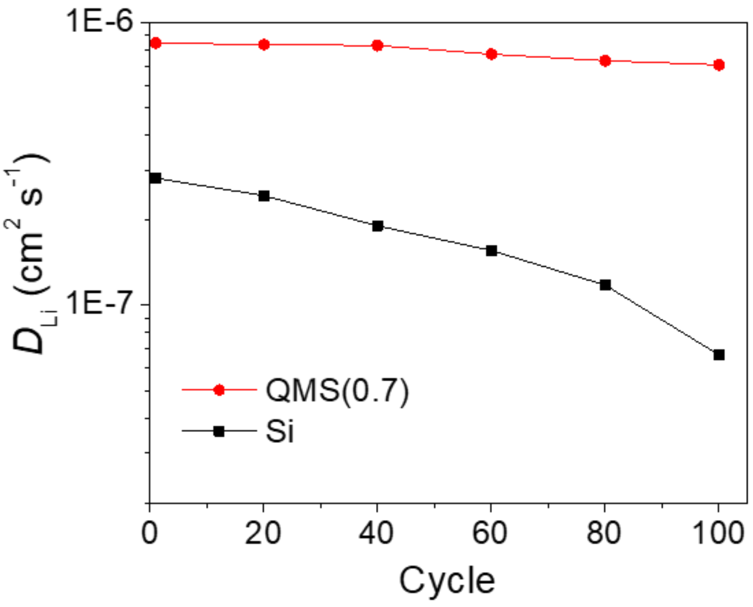

Supplementary Figure 11 | Diffusion coefficient calculation during cycles. Calculated diffusion coefficients of Si and QMS(0.7) electrodes after 1st, 20th, 40th, 60th, 80th and 100th cycles at 0.2 C.

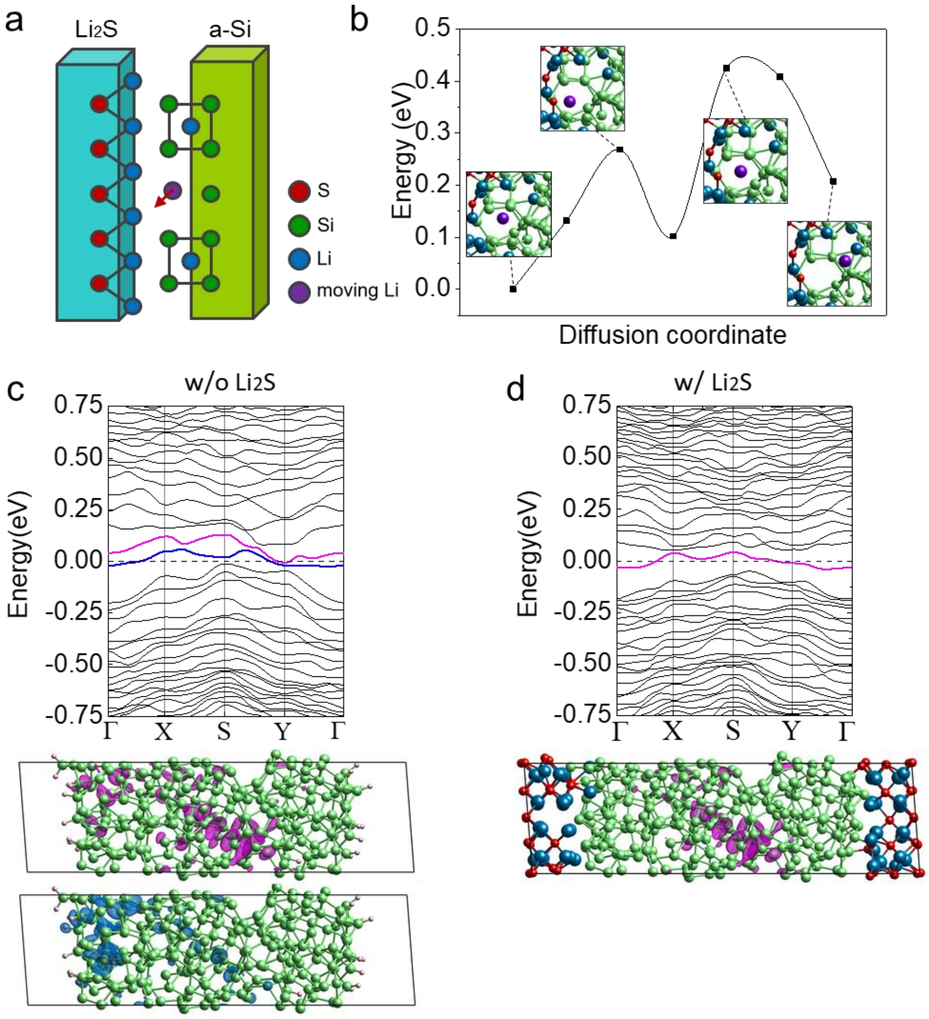

Supplementary Figure 12 | Formation of lithium sulfide inside quasi-metallic silicon. a Schematic illustration of Li diffusion path at an interface between lithium sulfide (Li_2S) and amorphous Si (a-Si). **b** Calculated diffusion energy barrier for Li-ion via the interface path. **c** Calculated band structure of the interface structure of H-passivated amorphous Si without Li_2S (Fermi level (E_f) set to zero) and charge density plot at CBM state (red line). White atoms on the silicon surface are hydrogen atoms. **d** Calculated band structure of the interface structure of a-Si with Li_2S and charge density plot at the state across the E_f (red line). Li_2S provides an electron to the unoccupied CBM state of internal amorphous Si, and QMS can have the metallic property by this charge transfer. The charge density plots demonstrate that the CBM state of (**c**) and the metallic state of (**d**) originates from the same internal Si. The other metallic band (blue line) can be explained with metallic surface states of the amorphous silicon, which has weaker metallic property[1,2] since the number of states is not related with bulk volume, but the red CBM can be proportional to bulk volume. Isosurface of the density is 0.0007 $e/Å^3$.

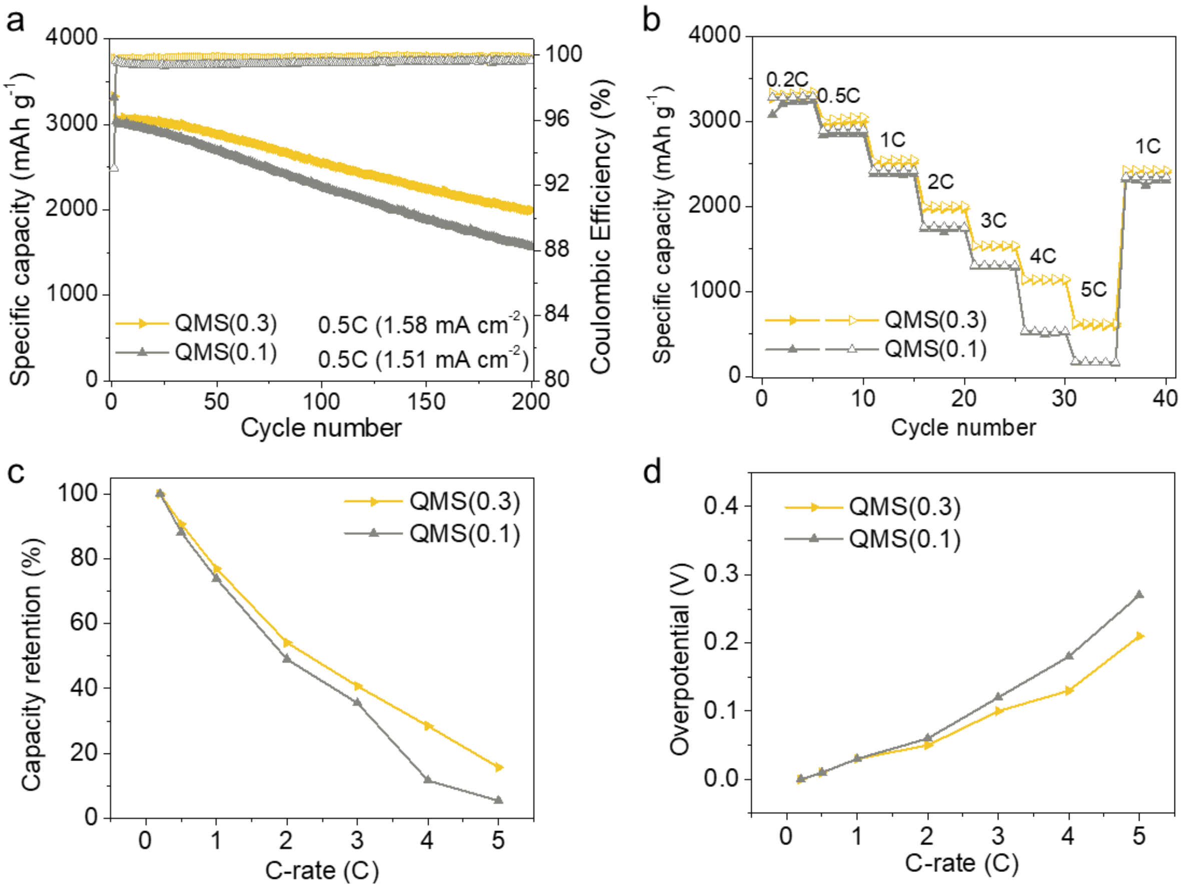

Supplementary Figure 13 | Electrochemical performances of QMS(0.3) and QMS(0.1) electrodes. a Capacity retention of QMS(0.3) and QMS(0.1) electrodes for 200 cycles along with corresponding Coulombic efficiency. **b−d** Specific capacities (**b**) with capacity retention plots (**c**) and Plots for estimated overpotentials at different C-rates (**d**) of QMS(0.3) and QMS(0.1) electrodes.

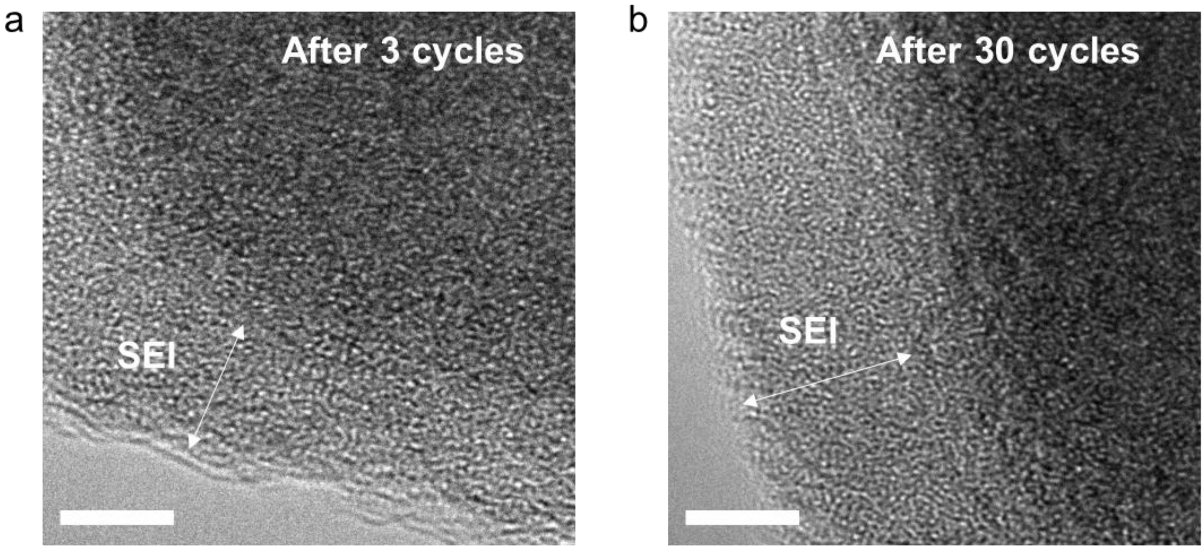

Supplementary Figure 14 | SEI layer of QMS(0.7) after cycles. a, b TEM images showing SEI layers of thickness less than 5 nm without facture and shell thickening after (**a**) 3 cycles and (**b**) 30 cycles of discharge/charge. Scale bars, 5 nm (**a,b**).

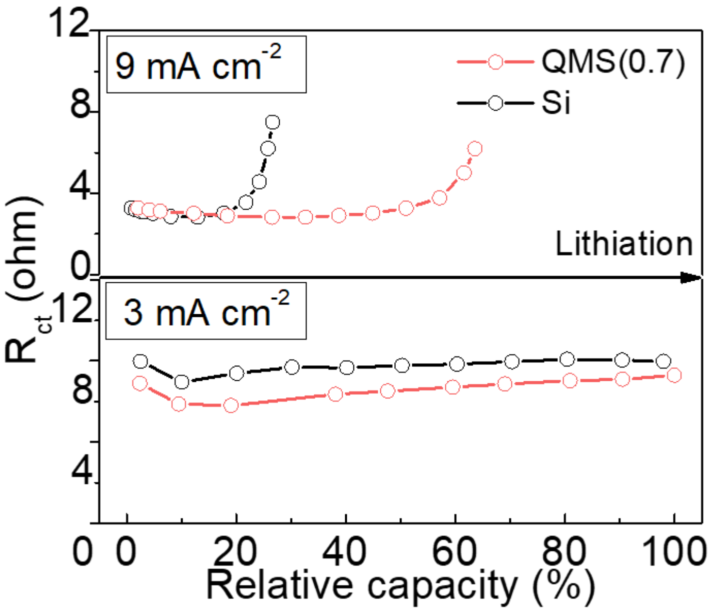

Supplementary Figure 15 | In-situ EIS Analysis of QMS(0.7) and Si electrodes. The plot of charge transfer resistance (R_{ct}) during in-situ EIS measurement for two electrodes at a different current density of 3 mA cm^{-2} and 9 mA cm^{-2}, corresponding to roughly 1 C and 3 C-rate.

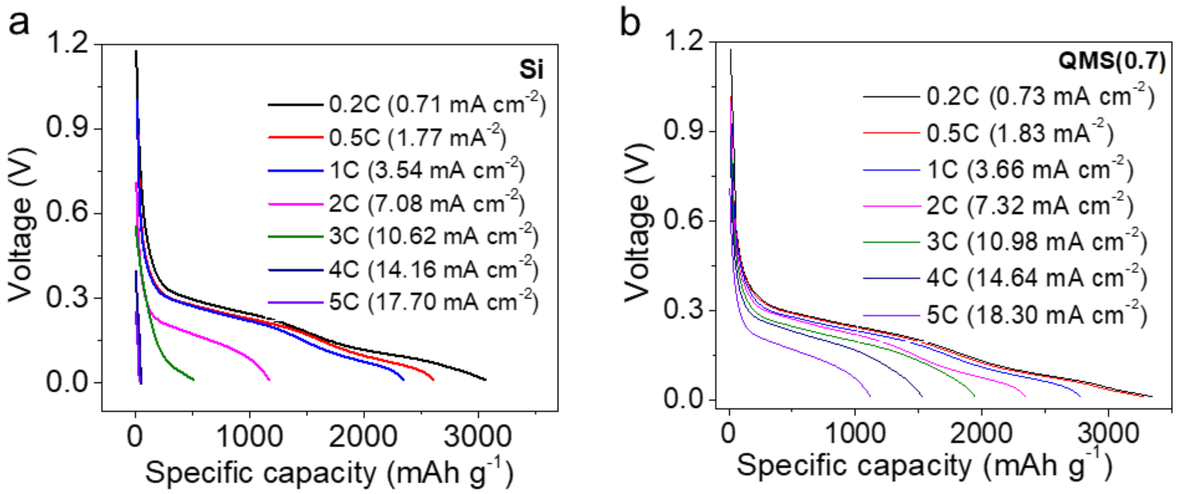

Supplementary Figure 16 | Overpotential analysis of QMS(0.7) and Si electrodes. a, b The voltage profiles of (**a**) QMS(0.7) and (**b**) Si electrodes at different C-rates from 0.2 C to 0.5 C, showing high polarization of Si electrode from at 2 C and stable lithiation behavior of QMS(0.7) electrode at 5 C without battery failure.

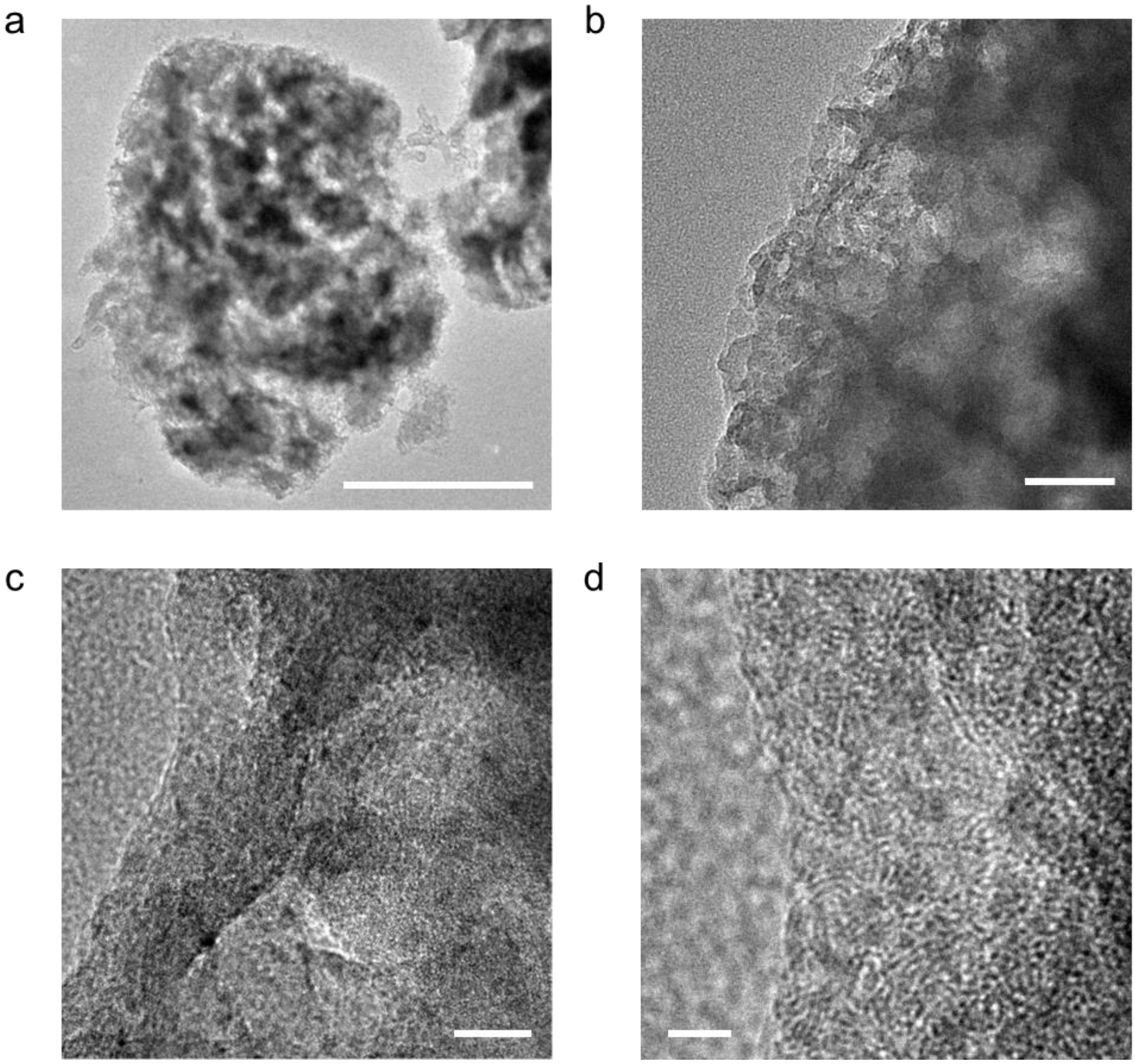

Supplementary Figure 17 | Post-mortem structural analysis on Si particle. a-d, TEM images of the Si particle after 50 cycles at different scales, showing the formation of extra pores on the structure with losing original structure and uneven outmost surfaces. Initially, existing mesopores became larger and merged with neighboring pores typically observed in porous Si materials. Yet, the unique structure of hollow and porous structure can sustain the initial framework without complete fracture or pulverization. When we previously introduce the carbon coating layers, its initial morphology was rather retained, indicating that the electric conductivity of bulk structure was closely related to structural integrity in case of microparticles. Scale bars, 1 μm (**a**); 50 nm (**b**); 10 nm (**c**); 2 nm (**d**).

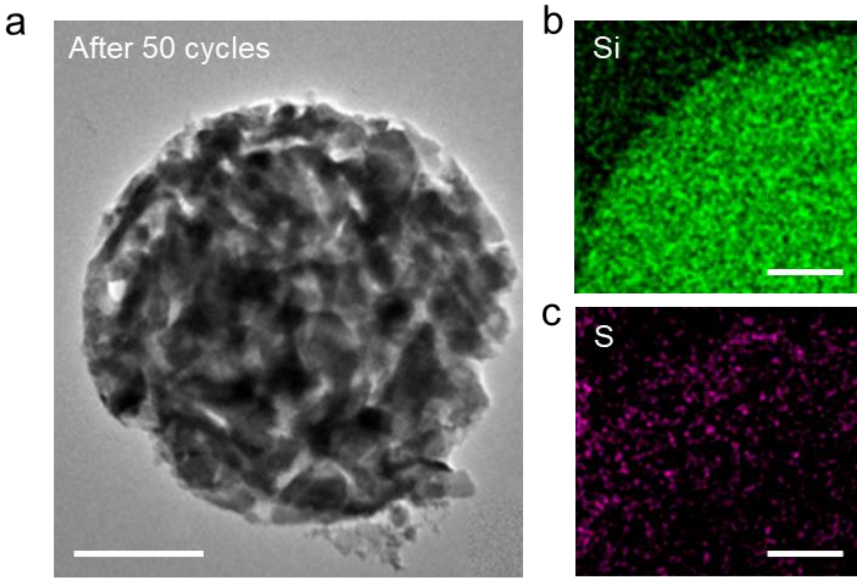

Supplementary Figure 18 | Post-mortem structural analysis on quasi-metallic silicon particle. **a-c** Structural deformation of QMS(0.7) sample after 50 cycles in its TEM image (**a**), and corresponding elemental maps (**b, c**) for Si and S of QMS particle, showing still uniform distribution of sulfur and sufficient pores that accommodate large volume changes even after cycles. Scale bars, 1 μm (**a**); 500 nm (**b, c**).

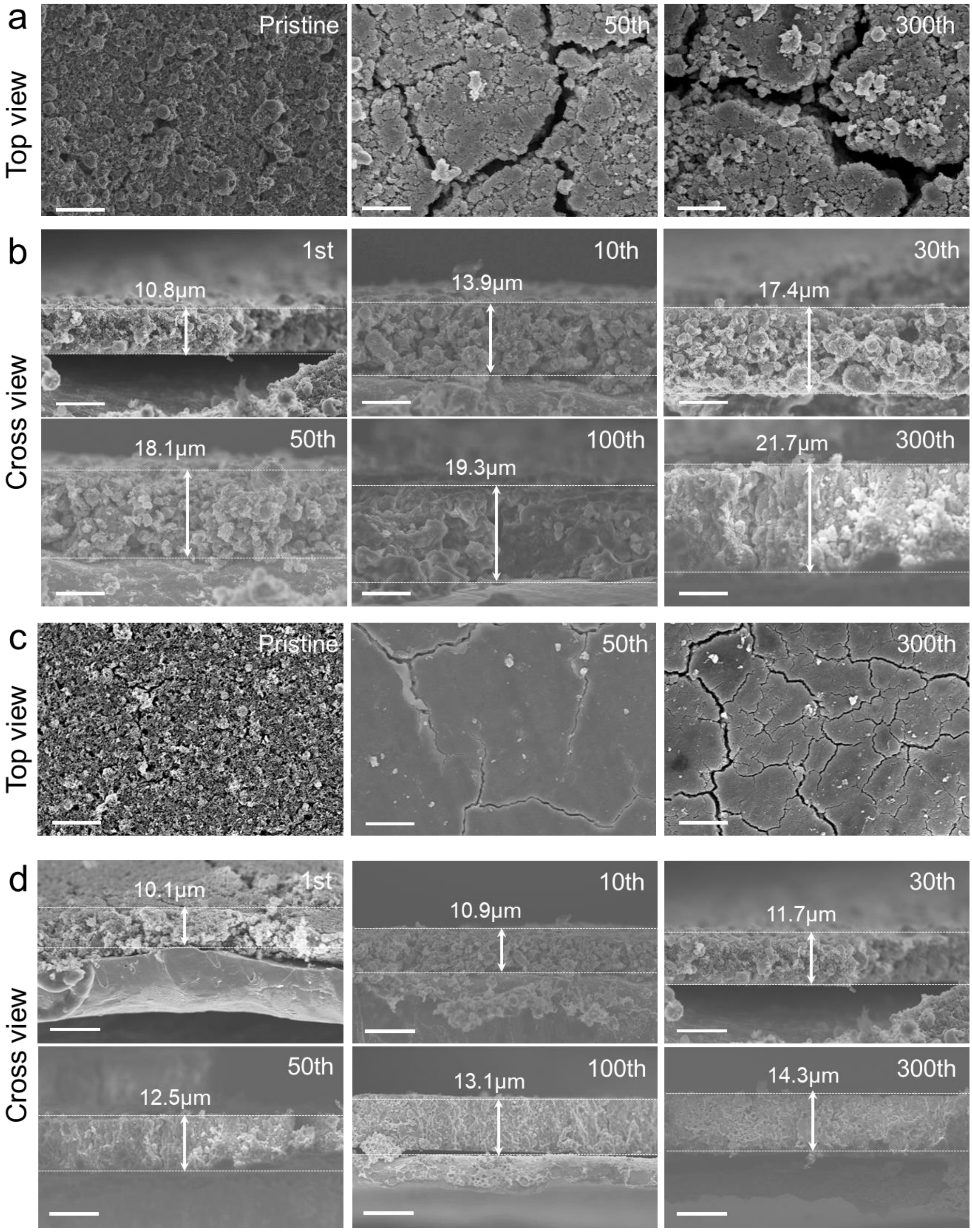

Supplementary Figure 19 | Electrode expansion of QMS(0.7) and Si electrodes. a-d Top-view and Cross-section SEM images of (**a, b**) Si and (**c, d**) QMS(0.7) electrodes at different stages of cycling during 300 cycles at 0.5 C. Scale bars, 10 μm (**a-d**).

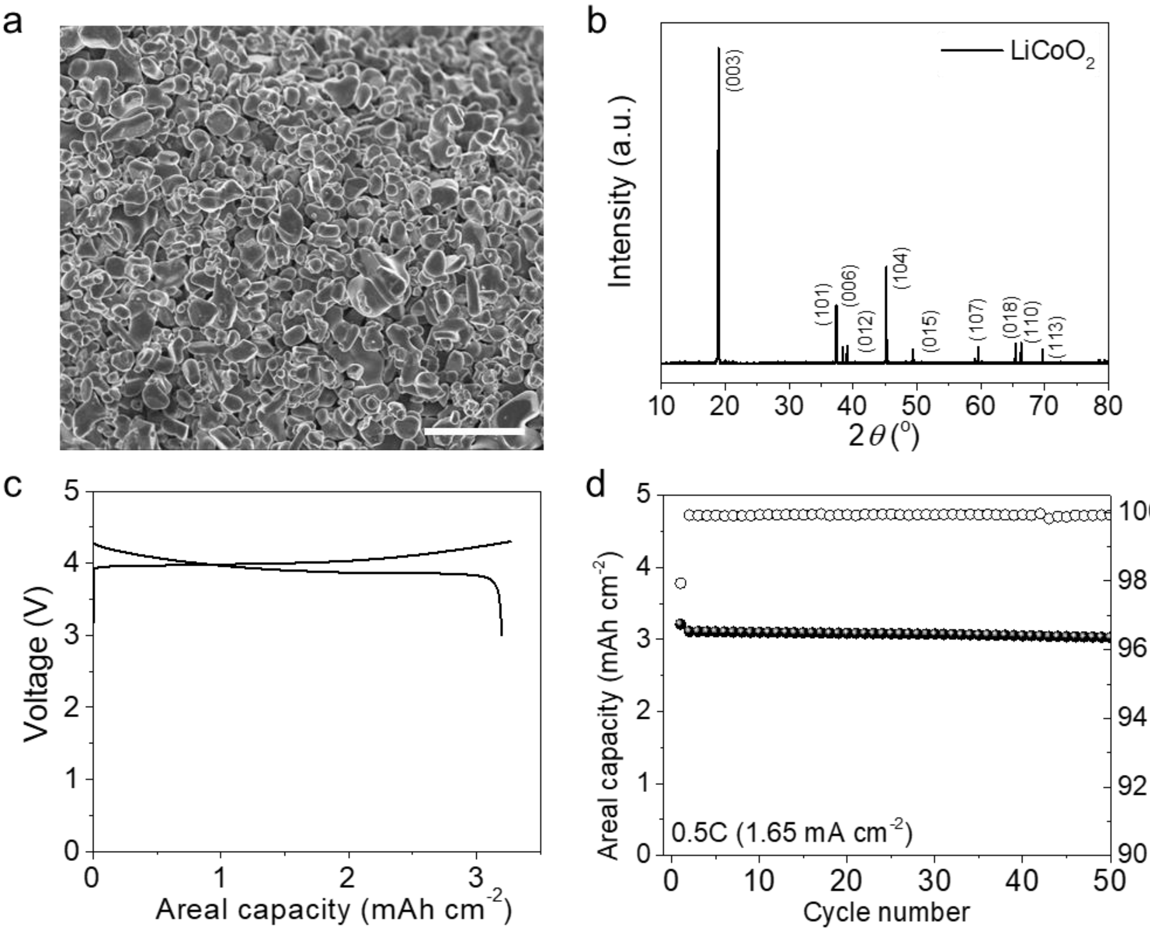

Supplementary Figure 20 | Physical and electrochemical properties of LCO cathode. a, **b** SEM image (**a**) and XRD pattern (**b**) LCO particles. **c, d** Voltage profile in the voltage window of 3–4.3 V at 0.1 C (**c**) and Capacity retention of LCO electrode for 50 cycles at 0.5 C (**d**). Scale bar, 20 μm (**a**).

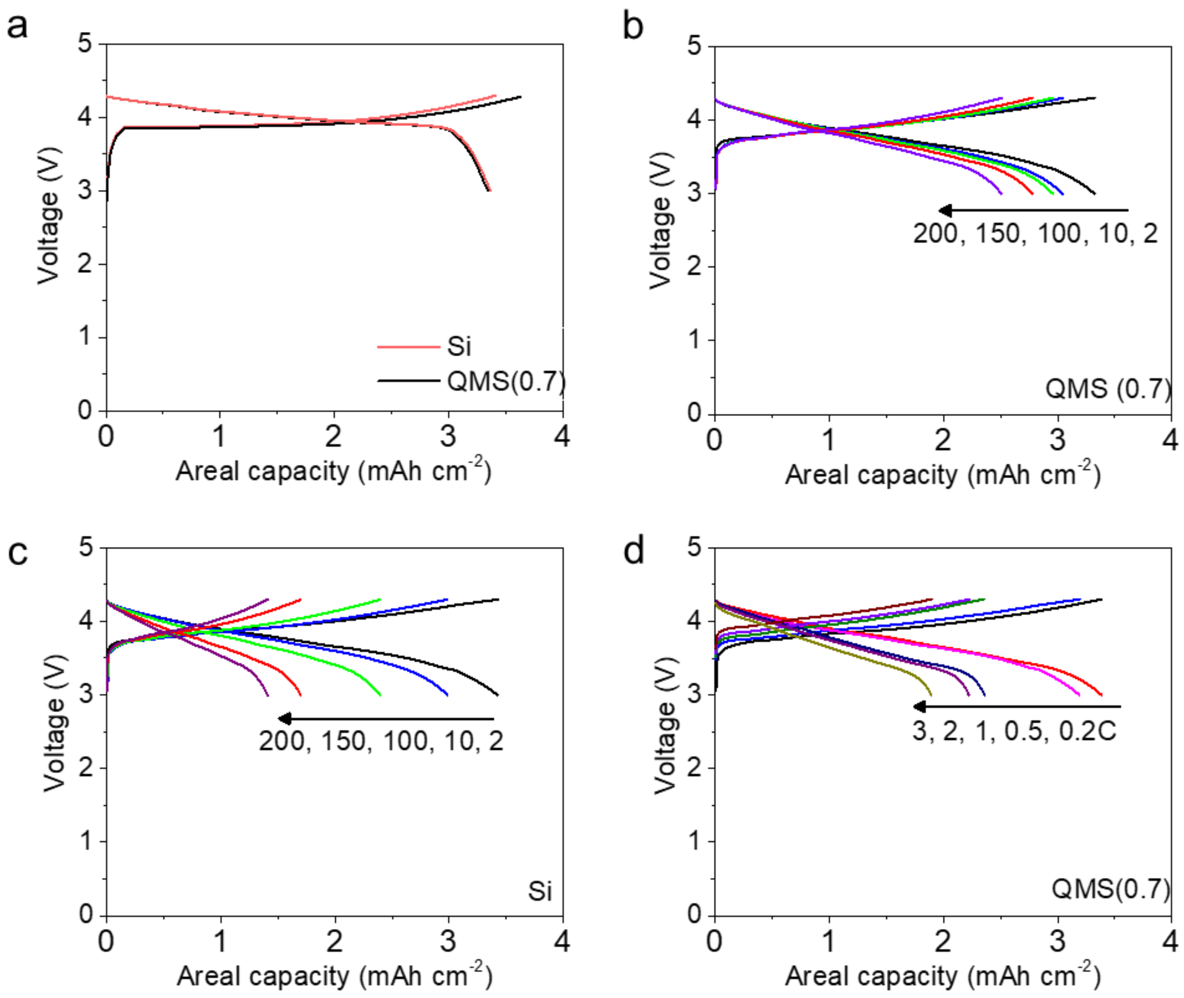

Supplementary Figure 21 | Electrochemical properties of QMS(0.7)/LCO and Si/LCO full cell. a Voltage profiles of QMS/LCO and Si/LCO full cells in the first cycle at 0.1 C. **b-d** Voltage profiles for 200 cycles at 0.2 C of (**b**) QMS/LCO and (**c**) Si/LCO full cells in the voltage window of 3.0–4.2 V. (**d**) Voltage profiles of QMS/LCO full cell at different C-rate of 0.2–3 C.

Supplementary Table 1. Comparison chart for recent progress of micron-sized Si anodes

Materials	Primary and/or Secondary particle size (μm)	Electrode composition (A:B:C)	ICE (%)	Areal capacity loading (mA h cm^{-2})	Initial reversible capacity (mA h g^{-1})	*X*, Capacity retention (%) after *Y*, cycles at Z, current density (A/g)			*X*, Capacity (mA h g^{-1}) and *Y*, capacity retention (%)[e] at *Z*, Max. discharge current density (A/g)			Ref.
						X	*Y*	*Z*	*X*	*Y*	*Z*	
**QMS(0.7)**	**ca. 3**	**80:10:10**	**92.5**	**~3.8**	**3,350**	**72**	**500**	**1.75**	**1,100**	**>35**	**17.5**	**This work**
Si		80:10:10	87.4	~3.3	3,080	<10	500	1.75	0	0	17.5	
Bulk porous Si@C	1.5<br>N/A[a]	70:0:30	92	3.4-4.5	~2,100	92.4	50	0.2	<1500	~50	2	4
mSi@OG[b]@ RGO[c]	1-5<br>N/A	80:10:10	78	~2.8	~2,500	~90	150	2.0	<500	<20	12	5
p-Si/C	<0.2<br>ca. 5.28	70:20:10	~75	2.8	~2,500	83	370	2.6	~700	~20	11	6
SiMP@SiC/ a-SiO_x/C/a-Li_2SiO_3	~1<br>N/A	70:10:20	77.7	~2.3	1,924	~75	100	0.1	1,439	N/A	0.1	7
Si/C	<0.1<br>ca.14.8	90:5:5	89.2	~2.85	620	0.75	500	0.06	~500	80	3.0	8

Si-MCS	<0.1<br>ca. 2	60:20:20	75	~2.44	~1,350	94	500	0.8	880	73	40	9
Si@Gr	1-3<br>N/A	90:10:0	93.2	~3.0	~3,300	~75	325	2.1	~350	~10	16.8	10
FeCuSi	<0.15<br>ca. 6.5	80:10:10 (Si)<br>97:1.5:1.5[d]	91<br>91.4	~1.3<br>3.44-3.48	1,287<br>420	~60	300	0.42	~252	N/A	0.42	11

Note: [a]Not applicable. [b]Overlapped graphene. [c]Reduced graphene oxide. [d]Graphite-blending electrode. [e]Capacity retention at max discharge current density compared with that measured at initial current density during rate capability test.

Supplementary Notes

Supplementary Note 1. Doping concentration control

The sulfur doping concentration in Si structure was controlled by different amounts of the sulfur source (magnesium sulfate) with the addition of extra aluminum and aluminum chloride in a precise proportion. Alternatively, changing amounts of sulfate at an excess aluminum and aluminum chloride is also acceptable to produce the same products of sulfur-doped Si. As differentiated from a conventional approach using the forced insertion of sulfur atoms into the crystalline Si structure (*i. e.* ion implantation), low-temperature sulfur fusion enables us to introduce the sulfur at the beginning of Si crystal formation during recrystallization of reduced Si and S seeds by metal-metal halide complex. As we described, the sulfur seeds are likely doped randomly into the Si structure in either configuration of substitution or chain formation. Otherwise, excessive amounts of sulfur that were not directly incorporated with Si remained as pure elemental sulfur in/outside of the Si structure.

Further, this non-equilibrium state of Si and S seeds might generate silicon sulfide structure of which conventional synthetic protocol requires high-temperature annealing in the presence of iron sulfide and carbon or thermal decomposition of reactive $(C_2H_5S)_4Si$ and sulfur at above 200 °C (ref. [3]). However, these compounds are unstable at the ambient atmosphere, thus leading to rapid dissociation of such compounds. In this context, when a stoichiometric amount of Si and S seeds are matched for the formation of silicon (di)sulfides, input sulfur sources are consumed for this unusual formation and eventually leached out during post treatments of aqueous hydrochloric acid. In addition to the elimination of isolated elemental sulfur by post heat treatment, sulfur loss from above speculation may give a decreasing trend in the sulfur amounts when input sulfur sources exceed a certain point. We take the sample with the highest sulfur amounts as QMS(0.7) and smaller amounts as QMS(0.3) and QMS(0.1). In order to validate an effect of the fused sulfur, electrochemical performances shown in the main figures are obtained from QMS(0.7) samples, while those of QMS(0.3) and QMS(0.1) are shown in **Supplementary Fig. 13**, having slightly improved performances when compared with undoped Si.

Supplementary Note 2. Energy density calculation

The energy density of batteries can be calculated by the following equation:

$$\begin{aligned} ED_v(Wh\,L^{-1}) &= areal\ cell\ capacity\ (mAh\ cm^{-2}) \times average\ voltage\ (V) \\ &\div electrode\ thickness\ (\mu m, anode + cathode + Separator) \\ &\times 10^4 (conversion\ factor) \end{aligned}$$

$$\begin{aligned} &ED_s(Wh\,kg^{-1}) \\ &\quad = cell\ capacity\ (mAh) \times average\ voltage\ (V) \\ &\quad \div electrode\ mass\ (mg, anode + cathode) \times 10^3 (conversion\ factor) \end{aligned}$$

where the areal cell capacity is ~3.3 mAh cm^{-2}, the electrode area is 1.54 cm^2, loading levels of the anode and cathode are 1.1 mg cm^{-2} and 23 mg cm^{-2}, respectively, the average voltage is 3.65 V, the electrode thickness in total is 101 μm (including 25 μm of PP separator), leading to volumetric energy density of 1193 Wh L^{-1} and 500 Wh kg^{-1} in the constructed full cell (QMS/LCO). The estimation of energy densities at high current density is not appropriate since the used LCO itself shows poor capacity retention at high C-rate (>3 C), for example, less than 60% at 3 C.

Supplementary References

1. Stuckelberger, M. et al. Review: progress in solar cells from hydrogenated amorphous silicon. *Renew. Sust. Energy Rev.* **76**, 1497-1523 (2017).
2. Nunomura, S. et al. Electronic properties of ultrathin hydrogenated amorphous silicon. *Appl. Phys. Express* **10**, 081401 (2017)
3. Haas, A. The chemistry of silicon-sulfur compounds. *Angew. Chem. Int. Ed.* **4**, 1014-1023 (1965).
4. Han, X. et al. Scalable engineering of bulk porous Si anodes for high initial efficiency and high-areal-capacity lithium-ion batteries. *ACS Appl. Mater. Interfaces* **11**, 714-721 (2019).
5. Zhang, X., Gua, R., Li, X. & Zhi, L. Scallop-inspired shell engineering of microparticles for stable and high volumetric capacity battery anodes. *Small* **14**, 1800752 (2018).
6. Jia, H. et al. A novel approach to synthesize micrometer-sized porous silicon as a high performance anode for lithium-ion batteries. *Nano Energy* **50**, 589-597 (2018).
7. Yang, Y. et al. Reaction-ball-milling-driven surface coating strategy to suppress pulverization of microparticle Si anodes. *ACS Appl. Mater. Interfaces* **10**, 20591-20598 (2018).
8. Xu, Q. et al. Watermelon-inspired Si/C microsphere with hierarchical buffer structures for densely compacted lithium-ion battery anodes. *Adv. Energy Mater.* **7**, 1601481 (2017)
9. Jeong, M.-G. et al. Self-rearrangement of silicon nanoparticles embedded in micro-carbon sphere framework for high-energy and long-life lithium-ion batteries. *Nano Lett.* **17**, 5600-5606 (2017)
10. Li, Y. et al. Growth of conformal graphene cages on micrometer-sized silicon particles as stable battery anodes. *Nat. Energy* **1**, 15029 (2016).
11. Chae, S. et al. Micron-sized Fe-Cu-Si ternary composite anodes for high energy Li-ion batteries. *Energy Environ. Sci.* **9**, 1251-1257 (2016).
